# An Upgraded, Highly Saturated Linkage Map of Japanese Plum (*Prunus salicina* Lindl.), and Identification of a New Major Locus Controlling the Flavan-3-ol Composition in Fruits

**DOI:** 10.3389/fpls.2022.805744

**Published:** 2022-03-04

**Authors:** Benjamín Battistoni, Juan Salazar, Wladimir Vega, Diego Valderrama-Soto, Pablo Jiménez-Muñoz, Ailynne Sepúlveda-González, Sebastián Ahumada, Isaac Cho, Claudio Sebastiano Gardana, Héctor Morales, Álvaro Peña-Neira, Herman Silva, Jonathan Maldonado, Mauricio González, Rodrigo Infante, Igor Pacheco

**Affiliations:** ^1^Instituto de Nutrición y Tecnología de los Alimentos (INTA), Universidad de Chile, Santiago, Chile; ^2^Programa de Doctorado en Ciencias Silvoagropecuarias y Veterinarias, Universidad de Chile, Santiago, Chile; ^3^Department of Plant Breeding, Centro de Edafología y Biología Aplicada del Segura, Consejo Superior de Investigaciones Científicas, Murcia, Spain; ^4^Dipartimento di Scienze per gli Alimenti, la Nutrizione, l’Ambiente, Università degli Studi di Milano, Milan, Italy; ^5^Departamento de Agroindustria y Enología, Facultad de Ciencias Agronómicas, Universidad de Chile, Santiago, Chile; ^6^Departamento de Producción Agrícola, Facultad de Ciencias Agronómicas, Universidad de Chile, Santiago, Chile; ^7^Centre for Genomic Regulation (CGR), Santiago, Chile

**Keywords:** major gene, QTL, epicatechin, catechin, Asian plum, *Prunus*, content, profile

## Abstract

Japanese plum fruits are rich in phenolic compounds, such as anthocyanins and flavan-3-ols, whose contents vary significantly among cultivars. Catechin (C) and epicatechin (EC) are flavan-3-ol monomers described in the fruits of this species and are associated with bitterness, astringency, antioxidant capacity, and susceptibility to enzymatic mesocarp browning. In this study, we aimed to identify quantitative trait loci (QTL) associated with the content of flavan-3-ol in Japanese plum fruits. We evaluated the content of C and EC in the mesocarp and exocarp of samples from 79 and 64 seedlings of an F1 progeny (<‘98–99’ × ‘Angeleno’>) in the first and second seasons, respectively. We also constructed improved versions of linkage maps from ‘98–99’ and ‘Angeleno,’ presently called single-nucleotide polymorphisms (SNPs) after mapping the already available GBS reads to *Prunus salicina* Lindl. cv. ‘Sanyueli’ v2.0 reference genome. These data allowed for describing a cluster of QTLs in the cultivar, ‘Angeleno,’ associated with the flavan-3-ol composition of mesocarp and exocarp, which explain up to 100% of the C/EC ratio. Additionally, we developed a C/EC metabolic marker, which was mapped between the markers with the highest log of odds (LOD) scores detected by the QTL analysis. The C/EC locus was located in the LG1, at an interval spanning 0.70 cM at 108.30–108.90 cM. Our results suggest the presence of a novel major gene controlling the preferential synthesis of C or EC in the Japanese plum fruits. This study is a significant advance in understanding the regulation of synthesizing compounds associated with fruit quality, postharvest, and human health promotion.

## Introduction

The *Prunus* genus comprises species with a high economic interest in agriculture, including peach, apricot, cherry, and the Japanese plum, among others (Carrasco et al., 2013; Ogah et al., 2014). The Japanese plum (*Prunus salicina* Lindl.) is a self-incompatible, diploid (2n = 16) species, originated in China, that produces an edible drupe (Topp et al., 2012; Wei et al., 2021). Also, it is known to be a rich source of phenolic compounds, whose types of molecules and their contents vary greatly among cultivars (Rothwell et al., 2013; Venter et al., 2014; Roussos et al., 2016).

Phenolic compounds are proposed to play a protective role in the interaction of plants with biotic and abiotic environments (Arici et al., 2014). Also, these are associated with human health benefits due to their antioxidant capacity, e.g., phenolic acids as neochlorogenic acid and flavonoids as anthocyanins and flavan-3-ols. Phenols can notably reduce the risk of diabetes, neurological disorders, and some types of cancer (Kumar and Goel, 2019). The antioxidant capacity of phenolic compounds is due to their chemical structure, which neutralizes the reactive oxygen species (Bagchi et al., 2003; Khoo et al., 2017; Ullah et al., 2019). Also, it has been demonstrated that these compounds can affect pigmentation, astringency, and bitterness of plant tissues, even if they are present in low concentrations (Soares et al., 2013). Because of their diversity, flavonoids have been used as models for analyzing a wide variety of genetic, epigenetic, cellular, biochemical, and evolutionary processes (Xu et al., 2015; Li et al., 2019; Zhu et al., 2020).

**FIGURE 1:**
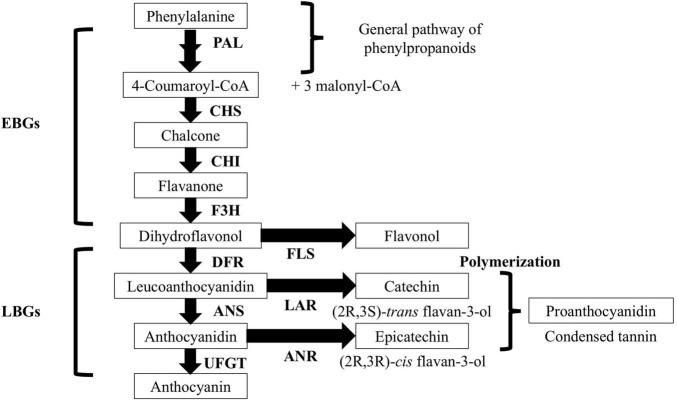
General diagram of the biosynthetic pathway of flavonoids in *Prunus salicina* Lindl. EBGs, early biosynthetic genes; LGBs, late biosynthetic genes; PAL, phenylalanine ammonia-lyase; CHS, chalcone synthase; CHI, chalcone isomerase; F3H, flavanone 3-hydroxylase; DFR, dihydroflavonol reductase; LAR, leucoanthocyanidin reductase; ANS, anthocyanidin synthase; ANR, anthocyanidin reductase; UFGT, UDP-flavonoid 3-*O*-glucosyltransferase; FLS, flavonol synthase. Modified from Dixon et al. (2013).

Flavan-3-ols are flavonoids whose structure consists of two phenyl rings and one heterocyclic ring. There are two main types of flavan-3-ols, catechin (C) and epicatechin (EC). C and EC are isomers that can form oligomers and polymers called proanthocyanidins (PAs); they are also known as condensed tannins (Koes et al., 2005; Dixon et al., 2013). The C and EC are biosynthesized by leucoanthocyanidin reductase (LAR) and anthocyanidin reductase (ANR) activities, respectively (Tanner et al., 2003; Xie, 2003; Figure 1). Leucoanthocyanidin is the common substrate for LAR and ANR and it is biosynthesized by converting dihydroflavonols into leucoanthocyanidins by dihydroflavonol 4-reductase (DFR). Additionally, before the catalytic action of ANR, the anthocyanidin synthase (ANS) must catalyze the conversion of leucoanthocyanidin into anthocyanidin (Wang et al., 2020).

The content of phenolic compounds (among them C and EC) is a quantitative trait that depends on various genetic and environmental factors (Venter et al., 2013; González et al., 2016). In the Japanese plum, cultivars, such as ‘Sun Breeze,’ ‘Laetitia,’ ‘African Delight,’ ‘Sapphire,’ ‘Ruby Red,’ ‘Ruby Crunch,’ and ‘Golden Japan’ showed equal or greater contents of C than EC (Nunes et al., 2008; Venter et al., 2014). However, the cv. ‘Sanhua’ presented higher contents of EC than C (Yu et al., 2021). Differences in the content of phenols have been described within the fruit, where the exocarp has a greater accumulation of these compounds than the mesocarp (Cabrera-Bañegil et al., 2020). Regulation of flavan-3-ols biosynthesis has been widely associated with a complex interaction between WD40, basic helix-loop-helix (bHLH), and R2R3-myeloblastosis (R2R3-MYB) transcription factors (TF), which form the MYB-bHLH-WD40 (MBW) complex (Gil-Muñoz et al., 2020). Also, it has been reported that wounding induces flavan-3-ol biosynthesis by activating the PA-related MYB regulator, DkMYB2 in persimmon (Akagi et al., 2010). Furthermore, the expression of the DkbZIP5 gene can activate DkMYB4 via an ABA signal, leading to a higher accumulation of flavan-3-ols (Akagi et al., 2012). Also, in peach (*Prunus persica* [L.] Batsch), PpMYB7, has been found to be associated with PpLAR1 activation but not PpANR, which in turn was regulated by PpbZIP5 in response to ABA (Zhou et al., 2015). In addition, the TFs PpMYBPA1 and Peace have also been described, which increase the content of flavan-3-ols in peach (Zhou et al., 2016). To date, knowledge about the genes controlling the contents of C and EC in the Japanese plum is limited. In this sense, Liu et al. (2020b) evaluated whole-genome sequences from *Prunus salicina* Lindl., indicating an expansion of PA-related MYB TF-coding genes, which suggests that a part of regulatory mechanisms associated with PA remain unexplored.

Quantitative trait loci mapping is a tool that identifies loci controlling traits of interest (Collard et al., 2005). Through QTL analysis, the loci controlling the ripening time, skin color, fruit mass, polar diameter, shape, blooming date, fruit development period, soluble solids content, malic acid, texture, and content of phenolic compounds have been described in the Japanese plum (Salazar et al., 2017, 2020; Valderrama-Soto et al., 2021). The studies mentioned above were performed by constructing linkage maps with GBS-based single-nucleotide polymorphisms (SNPs), called upon for mapping the obtained reads to peach v1.0 and v2.1 as reference genomes (Verde et al., 2013, 2017), and describing the synteny among species from the same genus. Recently, Liu et al. (2020a) have published the first reference genome of *Prunus salicina* cv. ‘Sanyueli,’ allowing for the construction of more dense and accurate linkage maps to identify QTLs controlling several traits, including the content of phenolic compounds, such as specific flavan-3-ols.

Therefore, in the current study, we associated genetic loci with the flavan-3-ol content of the mesocarp and exocarp of the Japanese plum fruits. Here, we reported a new version of the linkage maps of ‘98–99’ and ‘Angeleno’ (Salazar et al., 2017, 2020; Valderrama-Soto et al., 2021), now improved considering the release of a high-quality reference genome for *Prunus salicina* Lindl. (Liu et al., 2020a). These maps allowed for the precise identification and genetic mapping of a major locus controlling the C and EC contents of the Japanese plum F1 population (<‘98–99’ × ‘Angeleno’ >) by using the phenotypic and genotypic data.

## Materials and Methods

### Plant Material

Two Japanese plum cultivars (‘98–99’ and ‘Angeleno’) and 151 seedlings of the F1 progeny from their cross (<‘98-99’ × ‘Angeleno’>) were established at the ‘Rinconada de Maipú’ Experimental Station, Santiago, Chile (33° 30’ South, 70° 40’ West). The female parent, ‘98–99,’ is an early-medium maturing selection with red skin and yellow flesh. The male parent, ‘Angeleno,’ has a late-medium maturity, purple skin, yellow flesh, and excellent postharvest quality (Salazar et al., 2017, 2020). We included 79 and 64 seedlings from the F1 progeny, harvested in seasons 2015–2016 (T1) and 2016–2017 (T2), respectively. In T2, 15 individuals produced less than five fruits and, thus, were not comparable to the rest of the seedlings. As T2 samples were used as a comparison to verify the observed trends in T1, we decided not to sample these 15 seedlings. Samples employed in this study were the same as those from the studies reported by Salazar et al. (2017, 2020) and Valderrama-Soto et al. (2021). Fruits were harvested following the criteria reported by Contador et al. (2016), with chlorophyll absorbance index (IAD) values between 1 and 1.40 units and a firmness close to 40 N. After harvest, the fruits were frozen using liquid nitrogen and stored at –20°C until sample preparation.

### Flavan-3-ol Identification by Ultra High-Performance Liquid Chromatography-Diode Array Detector-Orbitrap-Mass Spectrometry (UHPLC-DAD-Orbitrap-MS)

Identification of flavan-3-ols was performed following Valderrama-Soto et al. (2021). The analysis was done on an Acquity UHPLC system (Waters, Milford, MA, United States), coupled with an eLambda DAD (Waters, Milford, MA, United States) and a High-Resolution Fourier Transform Orbitrap mass spectrometer, using the Extractive model (Thermo Scientific, Rodano, Italy), equipped with a HESI-II probe for ESI, operating in a positive mode and a collision cell (HCD). The MS data were processed using Xcalibur software (Thermo Scientific, Rodano, Italy). The peak identity was ascertained by evaluating the accurate mass, the fragments obtained in the collision cell, and by the online UV spectra (200–600 nm).

### Flavan-3-ols Quantification by High-Performance Liquid Chromatography-Diode Array Detector (HPLC-DAD) Method

From the frozen fruits, the exocarp (i.e., skin) was separated from the mesocarp (i.e., flesh). Each tissue had three replicates, where each replicate corresponded to an independent fruit. The amount of flavan-3-ols from the tissues was measured following the high-performance liquid chromatography-diode array detector (HPLC-DAD) technique, as reported by Peña-Neira et al. (2007). Extraction of flavan-3-ols was done from 3 g of skin and 10 g of flesh, which were ground in a laboratory mill (A 11 Basic, IKA, China) in liquid nitrogen until a homogeneous powder was obtained. Ground samples were macerated using 10 ml of 80% methanol for 20 min in ice. The extracts were filtered (0.22 μm) and stored in amber vials at 4^°^C in darkness until analysis. The HPLC-DAD analysis was done for the parents and the seedlings of the F1 population. The total amount (or absolute value) of each flavan-3-ol was measured in mg 100 g^−1^ of fresh weight (FW).

#### Phenotypic Data Analysis

The relative content of each flavan-3-ol was calculated by dividing the corresponding absolute content by the sum of the contents of flavan-3-ols found in the evaluated tissue. In T2, only the C and EC contents were determined. Also, the ratio between C and EC (C/EC) was calculated for each seedling in both seasons. Based on the C/EC values, the seedlings were classified depending on a threshold empirically determined according to the obtained results (refer to “Results” section “Catechin/Epicatechin Segregation”) since a clear gap in the relative content values was observed in both seasons. The C/EC threshold corresponded to the value which separated the seedlings based on the predominant content of C or EC in both the tissues. Flavan-3-ol contents in the F1 progeny were tested for normality by the Shapiro–Wilk test. The test of Levene was employed to determine the homogeneity of variance for each trait. Shapiro–Wilk and Levene tests with *p*-values greater than 0.05 indicate normal distribution and homogeneous variances, respectively. Histograms for each flavan-3-ol and scatterplot matrices were analyzed in each tissue in T1. Segregation analyses were performed according to the previously determined threshold, testing for the single gene model (3:1 or 1:1). Scatter plots, distribution, homogeneity of variance, and segregation analysis were done using the “pairs,” “hist,” “leveneTest,” and “chisq.test” functions, respectively, of the “stats” and “car” packages of R software v4.0.2 (R Core Team, 2020), using matrices generated in the tab-delimited text format (“.txt”).

### Linkage Map Construction

The linkage maps were constructed using the SNPs markers of 151 seedlings from the F1 population < ‘98–99’ × ‘Angeleno’>. Genotypic data obtained from DNA fragment libraries were generated by the genotype by sequencing (GBS) technology (Elshire et al., 2011), which was previously reported by Salazar et al. (2017). The genomic DNA was extracted following the protocol described by Doyle and Doyle (1987).

FASTQ files obtained from the GBS were processed with GBS pipeline v2 (Glaubitz et al., 2014)^1^. The sequences were aligned against *Prunus salicina* Lindl. cv. ‘Sanyueli’ v2.0 (Liu et al., 2020a)^2^, using the Burrows–Wheeler alignment tool v0.7.17 (Li and Durbin, 2009). The identified SNPs from each parent and seedling were stored in a VCF file format and finally filtered using the software, VCFtools v0.1.16 (Danecek et al., 2011). Only biallelic SNPs with a minimum allele frequency (MAF) of 0.05 and a maximum of 10% missing data were used. The VCF file with filtered SNPs was used for the linkage map construction. The names of the markers were assigned to represent their physical position in the Japanese plum reference genome. They consisted of a string of 11 characters, where the first two represent the corresponding super-scaffold (S1–S8) and the last eight, the physical position (in base pairs) of the SNP variation in the reference genome. The SNPs were manually converted to JoinMap format, filtering out those SNPs with missing data in the parents. Heterozygous SNPs in one of the parents were classified as segregation types < lmxll > or < nnxnp >, when the heterozygous parent corresponded to the female or male parent, respectively. The heterozygous SNPs in both the parents were classified as <hkxhk>. Map construction was performed using the JoinMap v4.1 (Ooijen, 2006), constructing the parental maps including <lmxll> or <nnxnp> SNPs, as appropriate. Also, <hkxhk> markers were included in the maps of both the parents.

Map construction was done using the regression mapping algorithm, where only the first or the second round was considered. Genetic distances were estimated through the Kosambi mapping function (Kosambi, 1943), with a maximum recombination frequency of 0.40. The SNPs with the logarithm of odds (LOD) values equal to or greater than 10 were grouped in each LG.

Considering the bimodal distribution of the C and EC contents, and the Mendelian segregation of the number of seedlings classified as C or EC (Table 1), a dominant metabolic marker was designed (C/EC). A new linkage map was constructed, adding the C/EC marker to the previous linkage map.

**TABLE 1:** Segregation analysis of the Japanese plum seedlings having a high content of catechin (C) or epicatechin (EC).

Season	Tissue	N° of seedlings	N° of C or EC seedlings		Expected segregation ratio	χ^2^
			C	EC		
T1	Flesh	79	48	31	1:1	4.57 (*p* = 0.05579)
					3:1	8.54 (*p* = 0.00347)
	Skin	79	48	31	1:1	4.57 (*p* = 0.05579)
					3:1	8.54 (*p* = 0.00347)
T2	Flesh	64	34	30	1:1	0.25 (*p* = 0.6171)
					3:1	16.33 (*p* = 5.31e^–5^)
	Skin	64	35	29	1:1	0.56 (*p* = 0.4533)
					3:1	30.08 (*p* = 4.14e^–8^)

*Segregation analysis was done using the chi-square test (χ^2^). C and EC contents were measured from the flesh and skin of the fruits, in two seasons (T1 and T2). Classification of C and EC was done according to a threshold based on a C/EC ratio of 0.30 and 0.70 in T1 and T2, respectively.*

### Quantitative Trait Loci Analysis

The QTL analysis was performed for each parent with the MapQTL version 6 (Ooijen, 2006) using the phenotypic data obtained from the HPLC-DAD analysis. Relative contents of each flavan-3-ol identified in T1 were included as traits. Additionally, a binary C/EC (C/EC-binary) trait was developed according to the estimated threshold from the C/EC ratio in T1 and T2. Seedlings with C/EC values less or greater than the threshold were classified as 1 or 100, respectively.

The SNPs were transformed to the double haploid (DH) format of the JoinMap, according to the pseudo-test cross method of MapQTL. Markers segregating from the cv., ‘98–99’ or ‘Angeleno’ were classified as “a” or “b,” respectively. Independent QTL analyses were performed for each tissue and season. The non-parametric Kruskal–Wallis and parametric interval mapping (IM) tests were used to determine significant marker-trait associations. The empirical LOD significance threshold (*p*-value < 0.05) for significant associations was determined after 1,000 permutations, according to the permutation test (PT) available in the MapQTL software. The QTL analyses were plotted using MapChart v2.32 (Voorrips, 2002).

## Results

### Identification and Quantification of Flavan-3-ols

Through ultra high-performance liquid chromatography-diode array detector-orbitrap-mass spectrometry (UHPLC-DAD-Orbitrap-MS) analysis, 21 phenolic compounds were identified from the Japanese plum fruits in T1 (Supplementary Figure 1). Flavonoid identification was done after extracting the skin and flesh samples of ‘Angeleno’ and ‘98–99’ by UHPLC-DAD-Orbitrap-MS (Supplementary Figure 2). Among the flavonoids, 6 compounds belonging to the flavan-3-ol family were identified; C, EC, proanthocyanidin B1 (PB1), proanthocyanidin A2 (PA2), and two non-identified flavan-3-ol dimers, designated as flavan-3-ol-a (F3L-a) and flavan-3-ol-b (F3L-b). In both parents, the contents of flavan-3-ols are shown only in T1. Also, none of the parents contained F3L-a or F3L-b, and PA2 was observed only in the flesh of ‘98–99’ (Supplementary Table 1). It is important to note that the content of flavan-3-ol compounds showed transgressive segregation, where some seedlings had less or higher contents of flavan-3-ols than that observed in the parents. Furthermore, some of the seedlings showed a lacking in one of the identified flavan-3-ols in the parents.

According to the results, flavan-3-ol content on the skin was higher than the values observed in the flesh (Supplementary Table 1). The parents showed total amounts of flavan-3-ols ranging from 7.75 to 10.41 mg 100 g^–1^ of FW and 14.65 to 19.40 mg 100 g^–1^ of FW in the flesh and skin, respectively. Also, the lowest contents in both the tissues were observed in the cv. ‘Angeleno.’ Regarding the F1 progeny in T1, the mean of the total content of flavan-3-ols was 2.46 and 20.69 mg 100 g^–1^ of FW in the flesh and skin, respectively. According to the Shapiro–Wilk tests, none of the evaluated traits showed a normal distribution. In T2, the absolute contents of C and EC were higher than those observed in T1 (Supplementary Table 1).

Further, the main compounds of the F1 progeny in both tissues were C and EC, whose sum corresponds to around 63–64% of the total content of flavan-3-ol in both flesh and skin. This trend was maintained in the parents, which showed that the amounts of C and EC were greater than 89% of the total flavan-3-ol content. Additionally, PA2 was identified only in 33.30% (flesh) and 3.60% (skin) of the seedlings and in the flesh of the parent ‘98–99.’ However, the content of C was higher in ‘98–99,’ while ‘Angeleno’ showed a higher content of EC.

**FIGURE 2:**
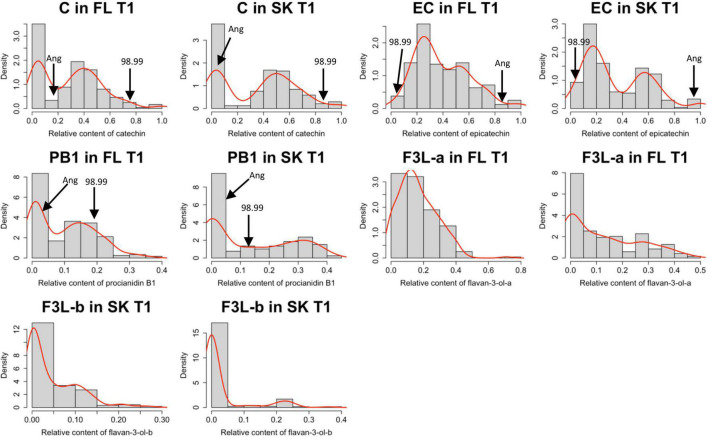
Data distribution of each flavan-3-ol was identified in the Japanese plum fruits. Each histogram shows the distribution of the relative content of each flavan-3-ol, relative to the total amount of flavan-3-ol observed. Flavan-3-ol contents were measured by the HPLC-DAD method in the flesh (FL) and skin (SK) of the Japanese plum fruits. The distribution of the relative contents is shown only in one season (T1). The mean values of the parents (‘98–99’ and ‘Angeleno’) for each trait are indicated with a black arrow. C, catechin; EC, epicatechin; PB1, proanthocyanidin B1; FL3-a and F3L-b, two flavan-3-ol dimers denoted as “a” and “b”.

Based on the contents of the identified flavan-3-ols, the relative content of each flavan-3-ol in T1 was calculated. Because of the low content of PA2 in the parents and the seedlings, this compound was excluded from the calculation of the relative values. The Shapiro–Wilk test indicates that the relative contents of flavan-3-ols presented a non-normal distribution (Supplementary Table 1). Also, based on the density curves, the relative contents of C and PB1 in both the tissues, and EC in the skin, showed a bimodal distribution (Figure 2).

### Catechin/Epicatechin Segregation

**FIGURE 3:**
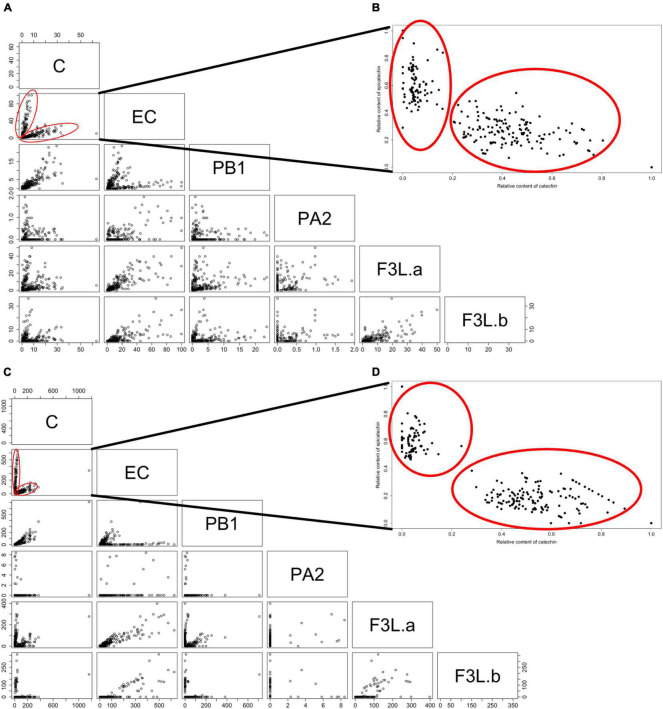
Pairwise scatter plots among the six flavan-3-ols identified in the F1 population < ‘98–99’ × ‘Angeleno’ > in the flesh **(A,B)** and skin **(C,D)**. **(A,C)** Correspond to the scatter plots using the data obtained by the HPLC-DAD method. **(B,D)** Correspond to the scatter plots between the relative contents of C and EC (Refer to section “Materials and Methods”). Data correspond to the flesh and skin of the Japanese plum fruits in one season (T1). C, catechin; EC, epicatechin; PA2, proanthocyanidin A2; PB1, proanthocyanidin B1; FL3-a and F3L-b, two flavan-3-ol dimers denoted as “a” and “b”. Red circles indicate the subpopulations observed among the evaluated seedlings.

To further analyze the contents of flavan-3-ols in the tissues on T1, pairwise scatter plots were constructed (Figure 3). Among the flavan-3-ols, pairwise using absolute values between C and EC showed a clear segregation pattern that suggests the presence of two subpopulations among the seedlings. The scatter plot with the relative values of C and EC in T1 allows for better visualization of the two subpopulations (Figure 3). Furthermore, it was impossible to observe a similar trend with the other flavan-3-ols, as with C and EC. According to these results, the C and EC contents in the fruits of the seedlings showed a segregation pattern that would allow for separating the F1 progeny into two subpopulations.

**FIGURE 4:**
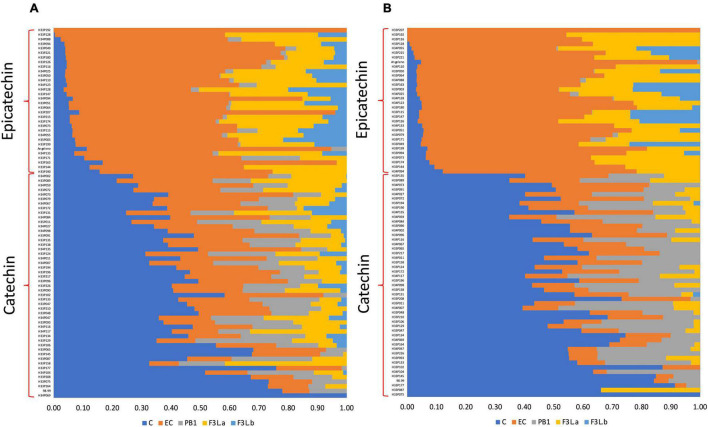
The relative content of flavan-3-ols identified in the flesh **(A)** and skin **(B)** of the Japanese plum fruits from an F1 progeny (<‘98–99’ × ‘Angeleno’) in T1. Data from the parents were included in each tissue. Data correspond to the relative content of each flavan-3-ol, in relation to the total amount of flavan-3-ols in the tissue, measured by the HPLC-DAD technique. C, catechin; EC, epicatechin; PA2, proanthocyanidin A2; PB1, proanthocyanidin B1; FL3-a and F3L-b, two flavan-3-ol dimers denoted as “a” and “b”.

To test the segregation pattern between C and EC contents among the seedlings, the C/EC ratio was calculated. According to the C/EC ratio in T1, in the flesh, we observed seedlings with a C/EC ratio less than 0.30 or greater than 0.50 (Figure 4). This difference was heavily marked in the skin, where it was possible to identify seedlings with a C/EC ratio less than 0.20 and more than 1.40. In T2, the C/EC ratio threshold in both the tissues was 0.70, maintaining a greater difference in the skin than in the flesh (Supplementary Figure 3). These results support a bimodal distribution of the C and EC contents. Furthermore, the number of seedlings classified as C or EC in both the tissues was tested against the single-gene model (Table 1). The number of seedlings belonging to the C or EC class in T1 was 48 and 31 in both the tissues, respectively. Segregation analyses showed that the C and EC contents fit with the 1:1 ratio (*p*-value > 0.05). Furthermore, the fruits of T2 showed 34 and 30 seedlings classified as C and EC in the flesh, while 35 and 29 seedlings belonged to C and EC in the skin, fitting with a 1:1 ratio (*p*-value > 0.05). Seedling classification across seasons was maintained in 100% of the cases in the skin and over 95% in the flesh.

### Linkage Mapping

Aligning and mapping GBS-based reads to *P. salicina* as the reference genome, we obtained 500,779 tags, of which 401,975 (80.27%) were aligned to a unique position, and 98,804 (19.73%) could not be aligned to the reference. Initially, 81,264 SNPs were obtained from the alignment; however, after applying filters (only two alleles, MAF > 0.05, and 10% as maximum missing data), we obtained 12,715 SNPs to be analyzed with the software, JoinMap v4.1. After discarding all the SNPs with identical segregation patterns, 1,126 SNPs were available for map construction.

**FIGURE 5:**
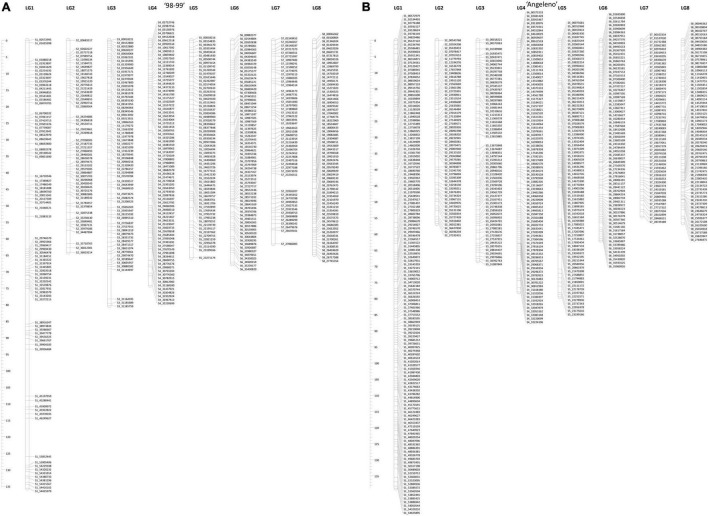
Linkage maps for each parent in the F1 Japanese plum population, ‘98–99’ **(A)** and ‘Angeleno’ **(B)**. On the left are shown the genetic distances (cM) for each map. On the right of each LG is the name of each SNP. The name of the SNPs indicates the corresponding LG and the physical position according to the *Prunus salicina* Lindl. reference genome.

From the 1,126 SNPs belonging to the eight *Prunus* linkage groups, 517 and 609 SNPs were segregated from the female (‘98–99’) and the male parents (‘Angeleno’), respectively (Figure 5 and Supplementary Tables 2, 3). The linkage maps spanned 614.28 cM and 595.31 cM for ‘98–99’ and ‘Angeleno,’ respectively (Supplementary Tables 4, 5). Distance between the markers ranged from 0.89 to 1.74 cM, while the maximum gaps on the LGs varied from 2.87 to 14.23 cM. Also, the LG1 was the largest in both the parents, showing the genetic distances of 135.62 cM (‘98–99’) and 138.78 cM (‘Angeleno’), respectively. Furthermore, the LG7 was the smallest, spanning 61.57 cM for ‘98–99’ and 54.52 cM for ‘Angeleno’.

### Quantitative Trait Loci Analysis and Integration of the Catechin/Epicatechin Metabolic Marker

Quantitative trait loci analyses with the updated version of the linkage map and the flavan-3-ol contents from the F1 population < ‘98-99’ × ‘Angeleno’ >, revealed significant QTLs only in ‘Angeleno,’ while ‘98–99’ did not show QTLs for the measured flavan-3-ols. Informative parameters about the QTLs observed from the parental ‘Angeleno’ are described in Supplementary Table 6.

We observed significant QTLs for each trait in both seasons, located only on the LG1, from 79.58 to 138.82 cM of the linkage map (Supplementary Table 6). All the QTLs observed in the flesh showed a lower LOD score and explained a lower percentage of phenotypic variance (PEV) than the skin. Smaller LOD scores were observed in PB1, F3L-a, and F3L-b, which showed values of 3.62–17.53, explaining between 19 and 63.20% of the PEV in the skin and flesh. Further, LOD scores of the QTLs for C and EC ranged from 19.32 to 35.13, with a PEV of 67.70–87.10%. Markers with the largest LOD scores included S1_43438292 (104.11 cM), S1_44899694 (106.23 cM), S1_46423283 (111.15 cM), and S1_47640923 (114.55 cM).

**FIGURE 6:**
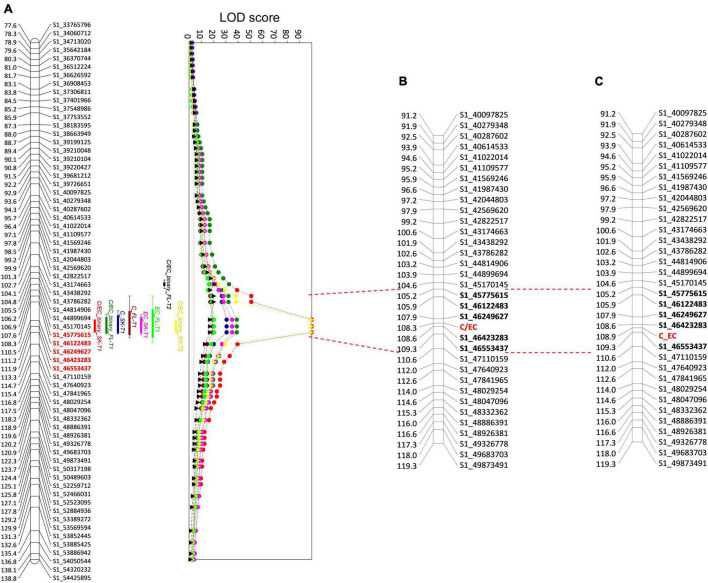
Quantitative trait loci (QTL) analysis of C and EC in ‘Angeleno’ **(A)** and linkage map including the C/EC metabolic marker in T1 **(B)** and T2 **(C)**. Each QTL corresponds to an independent trait from the skin (SK) and the flesh (FL) of the fruits in T1 (circles) and T2 (triangles). C, catechin; EC, epicatechin. QTLs for C and EC are shown only in T1, corresponding to the relative contents in the tissue. On the left side of the LG, the genetic position (cM) of each marker is indicated. On the right side, the name of each marker associated with a genetic position is indicated. The graph on the right indicates the LOD scores of the QTLs detected. The gray dotted line is the LOD score threshold for significant QTLs. Red lines extending from the QTL graph to the linkage maps with the C/EC marker denote the interval containing the markers (in bold) with the largest LOD scores. The name of the SNPs indicates the corresponding LG and the physical position according to the *Prunus salicina* Lindl. reference genome.

Following the segregation pattern in the F1 progeny in both seasons, an analysis with the C/EC-binary trait showed a QTL with a maximum LOD score of 38.93 in T1 and 20.24 in T2, explaining 89.70 and 76.70% of the PEV in the flesh, respectively. Furthermore, a QTL with a LOD score of 99.99 and a PEV of 100% was identified in the skin in both seasons (Figure 6). The QTLs detected in the flesh and skin span the same interval just like the other flavan-3-ols. Also, the maximum LOD score was observed in three consecutive markers in an interval of 1.41 cM (110.45–111.86 cM) in T1 and skin in T2. Nonetheless, the QTL of flesh in T2 was found in two additional markers located at 107.64 cM (S1_45775615) and 108.33 cM (S1_46122483). Altogether, the results from both seasons, the QTL associated with a higher content of C or EC is located between the markers, S1_45775615 and S1_46553437, spanning a genetic distance of 4.22 cM. The high explained variance of the detected locus indicates that on this precise interval of LG1, the variation in one gene modified the flavan-3-ol profile in the studied progeny.

In addition to QTL analysis, we included the C/EC metabolic marker in the linkage map constructed before (Figure 6). Marker C/EC was developed according to the tissue with the highest LOD scores, which corresponded to the skin in both seasons. Marker C/EC was mapped in LG1 in both the seasons, at 108.30 cM in T1, between the markers, S1_46249627 and S1_46423283, and at 108.90 cM in T2, between the markers S1_46423283 and S1_46553437, corresponding to those with the maximum LOD score for the trait, C/EC-binary. These results indicate that the locus, C/EC would be responsible for the content of C and EC in the tissues of the Japanese plum fruits since these compounds are the precursors of the other flavan-3-ols evaluated in this study.

## Discussion

### Flavan-3-ol Content of Japanese Plum Fruits

Japanese plum fruits are rich in phenolic compounds, such as phenolic acids, anthocyanins, flavonols, and flavan-3-ols families (Fanning et al., 2014). In this study, we measured the content of phenolic compounds (Supplementary Figure 1) in Japanese plum fruits of an F1 progeny and the parents (‘98–99’ and ‘Angeleno’) in two consecutive seasons. Here, we focused on the compounds belonging to the flavan-3-ol family (Supplementary Table 1). Of the identified flavan-3-ol compounds from the mesocarp and exocarp of the fruits, C, EC, PB1, and PB2 have been described in the previous studies (Venter et al., 2013; Yu et al., 2021). Also, the contents of the identified compounds in this study (C: 0.75–8.86 and 0.76–42.17 mg 100 mg^–1^ FW in the flesh and skin, respectively; EC: 1.04–14.01 and 0.97–66.35 mg 100 mg^–1^ FW in the flesh and skin, respectively; Supplementary Table 1) agree with the contents of flavan-3-ols in the Japanese plum fruits reported in previous studies (Nunes et al., 2008; Venter et al., 2014). Compared to other *Prunus* species, we found that the abovementioned C and EC content range in the parents and the studied F1 outperform the contents of C and EC found in sweet cherry (*Prunus avium* L.), a species known to have a high content of flavonoids. Regarding this, Kelebek and Selli (2011) reported that the contents of C and EC range between 2.92–9.03 and 6.33– 4.84 mg 100 mg^–1^ FW, respectively, in whole fruits, i.e., skin and flesh mixed in the same sample; Hayaloglu and Demir (2016) reported that the contents range between 0.93–2.72 and 5.27–14.67 mg 100 mg^–1^ FW for C and EC, respectively. The high content and variation in the contents of C and EC found in the studied progeny suggest that the Japanese plum is an important species for the genetics studies of the secondary metabolite profile.

Both the F1 progeny and the parents showed greater contents of flavan-3-ols in the skin than in the flesh in T1 and T2. Flavan-3-ol contents agree with the total amount of phenolic compounds of the Japanese plum fruits, which have higher accumulation in the skin than in the flesh (Cabrera-Bañegil et al., 2020; Valderrama-Soto et al., 2021). Also, we observed a greater accumulation of C and EC in T2 than T1. The differences among the seasons and fruit tissues would confirm the influence of the environment on the contents of phenolic compounds of the plant species (Sahamishirazi et al., 2017), which could lead to a higher or lower content of some phenols in the seedlings, compared to the parents.

The flavan-3-ol content is a quantitative continuous trait, as observed in the data distribution (Figure 2). In this study, the relative contents of C, EC, and PB1 showed a bimodal distribution in both tissues. Multimodal distribution of continuous quantitative traits has been described previously. In *Prunus persica*, two F2 populations (<‘Contender’ × ‘Ambra’> and <‘N.J. Weeping’ × ‘Bounty’>) showed a trimodal distribution for the maturity date, showing a correlation coefficient of 0.92 between years (Pirona et al., 2013). As with the maturity date in peach, and despite the relative contents of C, EC, and PB1 being a quantitative trait, their distributions suggest a Mendelian segregation pattern in the F1 progeny < ‘98–99’ × ‘Angeleno’ >. Only the scatter plot between C and EC showed clear segregation among the seedlings highlighted when evaluating the relative contents. According to the C/EC threshold in T1 and T2, the number of seedlings classified as C or EC in both the tissues agreed with a 1:1 segregation pattern (Table 1). Following the results, the differences in the C and EC contents among the seedlings and the parents suggest a genetic difference controlling the preferential synthesis of one of these compounds in the Japanese plum.

The contents of C and EC in the Japanese plum have been determined in the previous studies. Nunes et al. (2008) measured the content of flavan-3-ols in ‘Golden Japan’ Japanese plum fruits observing a higher content of C than EC in both the skin and flesh. Also, Venter et al. (2014) evaluated six cultivars and four selections of the Japanese plum at two fruit maturity stages (unripe and ripe). From those analyses, they observed a greater content of C than EC in most of the analyzed fruits, and only one selection showed a greater content of EC than C. Additionally, Yu et al. (2021) evaluated the cultivar, ‘Sanhua,’ which presented a higher EC amount than C. Here, we evaluated the selection, ‘98–99’ and the cultivar, ‘Angeleno.’ The former showed a higher content of C than EC, and the latter exhibited a higher content of EC. In addition to the segregation pattern of the F1 progeny (‘98–99’ × ‘Angeleno’), the previous results reinforce the idea that a genetic cause underlies the difference between the C and EC contents of Japanese plum fruits. The C and EC compounds are involved in fruit quality due to their influence on sensory traits, such as astringency and bitterness (Dixon et al., 2013). Also, these compounds are associated with health benefits for consumers due to their antioxidant capacity. Despite the similar chemical structure of C and EC, they show differences in essential aspects of food quality, such as bioaccessibility and bioavailability (Baba et al., 2001; Yu et al., 2021). Additionally, C and EC compounds influence the susceptibility to enzymatic browning, where EC is preferentially used as a substrate by the polyphenol oxidase (PPO) compared to other phenols, including C (Li et al., 2021). This suggests that fruits with higher contents of EC would be more sensitive to enzymatic browning than those with higher contents of C.

### Construction of a Linkage Map Using the *Prunus salicina* Reference Genome

The genotypic data employed in this study has been used for reconstructing the previously reported linkage maps, in which the genomes of *Prunus persica* v1.0 and v2.1 were employed as references (Salazar et al., 2017, 2020; Valderrama-Soto et al., 2021). We updated previous linkage maps of ‘98–99’ and ‘Angeleno,’ using *P. salicina* ‘Sanyueli’ as reference genomes (Liu et al., 2020a) instead of peach. Previous parental linkage maps accounted for a total of 981 (Salazar et al., 2017) and 1,207 (Salazar et al., 2020) SNPs, while here, we reported 1,126 high-quality SNPs.

For the parental ‘98–99,’ the previous genetic distances were 688.80 and 557.32 cM, while ‘Angeleno’ showed 647.03 and 576.51 cM, which are the higher values corresponding to those constructed using the *P. persica* v1.0. It is important to note that the second version of the linkage map included 41 SSR markers, which explain the differences in the genetic distances from the first version. The integration of more markers allowed increased linkage saturation, leading to a lower recombination frequency, reducing the final genetic distances (Salazar et al., 2020). The genetic distances of the new linkage maps are 614.28 cM (‘98–99’) and 595.31 cM (‘Angeleno’), which are similar to those obtained in the second version. Despite the previous versions showing collinearity among the species from the same genus, we observed that choosing a reference genome of the same species could lead to a more accurate estimation of genetic distances without adding new markers.

Genetic linkage maps for the Japanese plum cultivars have been published previously. First, Vieira et al. (2005) used 56 and 84 AFLP to develop maps of ‘Chatard’ and ‘Santa Rosa,’ respectively. These maps accounted for the genetic distances of 905.50 and 1,349.60 cM. Later, Salazar et al. (2017) published linkage maps from the same genotypes used in this study and were updated by Salazar et al. (2020). Carrasco et al. (2018) also reported linkage maps for the cultivars, ‘Angeleno’ (588 cM) and ‘Aurora’ (490 cM), accounting for a total of 1,441 high-quality SNPs. Later, Zhang et al. (2020) published linkage maps for ‘09–16’ and ‘Fortune’ using a total of 3,341 SNPs. All the studies mentioned employed the peach reference genome instead of the Japanese plum. Here, we used the chromosome-level draft of *P. salicina* (Liu et al., 2020a), which allowed us to obtain a greater alignment compared to the former. We aligned 80.27% of the total reads obtained from sequencing, while those reported by Salazar et al. (2017) and Carrasco et al. (2018) only aligned 42.90 and 48.60% of the total reads obtained from the sequencing, respectively. In addition, the present linkage map allowed for the reduction of the mean distance between SNPs, compared to those observed by Salazar et al. (2017, 2020), resulting in a decrease from 1.39 to 1.20 cM in ‘98–99’ and 1.05 to 0.99 cM in ‘Angeleno’. Additionally, we reduced the mean gap observed in ‘98–99’ from 7.34 to 6.32 cM, while ‘Angeleno’ maintained the mean gap of 4.80 cM. Carrasco et al. (2018) presented some SNPs showing discordance with their corresponding physical positions. Here, we filtered out all those single SNPs that did not match the physical position, generating uncertainty regarding their quality, probably due to errors in the assembly of the reference genome or genotyping errors (i.e., SNP call). Even by discarding such SNPs, we obtained a higher density in our maps compared to the results of Salazar et al. (2017, 2020) and Carrasco et al. (2018). These results indicate a clear improvement concerning the previously published linkage maps of ‘98–99’ and ‘Angeleno’ (Salazar et al., 2017, 2020), suggesting that the use of a reference genome of the same species (*Prunus salicina* Lindl.) avoids the loss of information due to specific points of sequence divergence among species, despite the collinearity shown by previously cited studies.

### Quantitative Trait Loci Analysis of the Catechin and Epicatechin Contents

In this study, we reported a cluster of QTLs located in LG1, which indicates the existence of a locus controlling the synthesis of the flavan-3-ols present in the Japanese plum fruits. Furthermore, the main QTLs are related to C and EC contents, which suggests that the locus would be associated with the preferential synthesis of C or EC in the fruit tissues and not with the total amount of flavan-3-ols. The above can be explained because PAs (i.e., PB1, PA2, FL3-a, and F3L-b) are produced by the polymerization of C and EC, and their difference would be caused by the changes in the content of the flavan-3-ol monomers (Dixon et al., 2013).

Results from the QTL analysis agree with those observed in the phenotypic data, which suggest a bimodal pattern in the C and EC contents (Figure 2). Additionally, QTLs were only observed in the parental ‘Angeleno,’ which indicates that the genetic variation involved in the C/EC phenotype is heterozygous in this cultivar and homozygous in ‘98–99.’ It is important to notice that the segregation analysis in T1 showed an identical number of seedlings classified as C or EC. However, the PEV of the QTL in the flesh (89.70%) and skin (100%) were different despite the samples of each seedling were from the same fruits. Regarding the data obtained from the HPLC, in T1, two seedlings from the flesh were classified conversely from those observed in the skin. Additionally, some seedlings showed a different classification in T1 and T2, which could be because of errors in the measurement of the samples or due to the effect of environmental factors, since they are mainly responsible for variations in the contents of phenolic compounds (Arici et al., 2014). Nonetheless, more than 95% of the seedlings showed identical classification among seasons, which agrees with the QTL analysis in the second season. In T2, the QTL observed in the skin was maintained in the same position as T1, whilst in flesh, two markers in the lower genetic distance, in comparison with the skin, corresponded to the highest LOD scores (Figure 6). These results are associated with the difference in the C/EC classification between T1 and T2.

The metabolic marker C/EC was mapped in the LG1 of ‘Angeleno’ at 108.30 cM in T1 and 108.90 cM in T2, between the markers with the maximum LOD scores observed in the traits C, EC, and C/EC-binary. These results consolidate the hypothesis that there is a genetic regulation of the synthesis of C and EC in Japanese plum fruits located in the lower part of chromosome 1, between markers S1_46249627 and S1_46553437.

Previous studies of QTL analysis on *Prunus salicina* Lindl. have been published by our team, evaluating the productive and postharvest traits (Salazar et al., 2017, 2020). Valderrama-Soto et al. (2021) have investigated QTLs associated with the phenolic composition in the Japanese plum, detecting QTLs for total phenols, total flavonoids, antioxidant capacity, and some anthocyanin compounds in the LG1, 3, 4, 5, 7, and 8. In contrast, in that study, the QTLs for the total content of flavan-3-ols were located in the LG5 and 8, which could be explained because the QTLs identified in the present study were related to the changes in the flavan-3-ols profile and not to the total content. Additionally, they indicated that flavan-3-ols correlate with the higher antioxidant capacity of the fruits, which supports the importance of these compounds in human health (Yu et al., 2021).

According to the C/EC locus in the linkage map, the causative mutation conferring a preferential synthesis of C or EC is in a 0.70 cM interval between the markers, S1_46249627 and S1_46553437, corresponding to a physical distance of 303,810 bp. Despite the suggested interval, it is necessary to do a fine-mapping for this new locus to determine this novel major gene controlling the C/EC ratio in the Japanese plum fruits. Regarding the C and EC contents, in peach, it has been previously described that the TF PpMYB7 activates the gene encoding LAR but not ANR (Zhou et al., 2015; Figure 1). These results suggest that there exists a genetic mechanism that activates the synthesis of C or EC in the fruits. Additionally, a DkbZIP5 induces an upregulation of DkMYB4 in persimmon, which results in a greater PA biosynthesis (Akagi et al., 2012). Furthermore, MdMYB9 and MdMYB11 promote the synthesis of anthocyanins and PAs by directly binding to the promoters of genes coding ANS, ANR, and LAR in apples. Also, MdbHLH3 TF binds to the promoters of MdMYB9 and MdMYB11 positively regulating their transcription (An et al., 2015).

In this study, we showed the results of flavan-3-ol profiles of the Japanese plum fruits of an F1 progeny. Although the phenolic composition is a complex trait regulated by several genes, the C/EC content behaves as a monogenic-controlled trait. This could indicate that a major gene controls their synthesis. Furthermore, the QTL analysis showed the existence of a locus in the LG1 of the parental ‘Angeleno,’ which explains up to 100% of the phenotypic variation. Altogether, the results from this study and those on peach, persimmon, and apple, indicate that variations in the C/EC ratio in the Japanese plum fruits could be regulated by TF belonging to MYB, bHLH, or bZIP families. According to the gene prediction made by Liu et al. (2020a), available in https://www.rosaceae.org/Analysis/9450778, in the locus of C/EC, there are a total of 62 genes (Supplementary Table 7), which could be candidates for controlling the synthesis of C or EC. Analyzing the GO terms of the genes within the interval, the software, REVIGO (Supek et al., 2011)^3^ indicates that 31, 12, and 39 terms are involved in the biological process, cellular component, and molecular function, respectively. It is important to note that from these categories, 24.56% of the GO terms refer to the biosynthetic process and 13.62% to the transmembrane transport, which could be involved in the biosynthesis and transport of flavonoids.

Within the suggested interval, there are three predicted genes described as flavonol synthase, which are involved in the flavonoid pathway (Figure 1). Additionally, there are two genes encoding for an ethylene-responsive transcription factor (ERF), which could be involved in the C/EC trait since ethylene is a growth regulator involved in the biosynthesis of anthocyanins (Gao et al., 2020). Nonetheless, it has been observed that in the Japanese plum, C and EC contents are not directly affected by the exogenous ethylene (Niu et al., 2017). Genetic variations could be responsible for a novel genetic regulation associated with flavan-3-ols, such as C and EC since the Japanese plum is a climacteric species. Three genes encoding for WAT1-related proteins are present in tandem within the C/EC locus. The WAT1 proteins participate in the auxin export from vacuoles, suggesting a key role in the intracellular auxin homeostasis (Ranocha et al., 2013). Auxins are indispensable growth regulators involved in plant cellular growth, which could also be associated with adaptation mechanisms controlling C and EC in the fruit tissues in response to environmental stresses. Further analyses are required to determine candidate genes possibly responsible for the C/EC ratio in the Japanese plum fruits. In this sense, a *de novo* assembly of the chromosomal region containing the genetic variation in ‘Angeleno’ would be an initial step for the precise identification of the variants (e.g., SNP, InDel) causing the preferential synthesis of C or EC. Crossing the variants’ positions with annotated genes in the region could shorten the list of candidate genes. The so-called variants should also be tested in other F1 progenies from ‘Angeleno’ crossed with other varieties, to gain knowledge about the genetic control of this trait in different genetic backgrounds of the Japanese plum.

Gaining insight into the synthesis of C and EC in fruits is of special interest since they are involved in many important fruit traits. In this sense, results reported in this study open up possibilities for the fine mapping of the locus containing the gene responsible for promoting the content of C or EC in the Japanese plum fruits, which could even be tested on other progenies of the current species. The precise determination of the locus would allow for identifying the allele of the gene responsible for the preferential content of C or EC in this species, allowing for a further genetic characterization using other Japanese plum segregating progenies.

## Data Availability Statement

The data presented in the study are deposited in the Genome Database for Rosaceae repository, https://www.rosaceae.org/publication_datasets, accession number tfGDR1054.

## Author Contributions

BB performed the bioinformatic analyses, linkage mapping, phenotypic data analysis, QTL analysis and wrote the first draft of the manuscript. IP developed the concept of the work, applied for funding, coordinated experimental work and data analysis, and supervised manuscript writing. RI developed the crosses and F1 populations and maintained them on the field in good conditions. JS coordinated harvest, quality analysis, and fruit sampling. DV-S, AS-G, WV, IC, ÁP-N, and HM participated in the quantification and analyses of the compounds. CG performed MS compound identification. HS and JM participated in the bioinformatic analysis. RI, PJ-M, MG, HS, JM, and JS participated in the critical revision, editing, and preparation of the final version of the manuscript. All the authors contributed to the article and approved the submitted version.

## Conflict of Interest

The authors declare that the research was conducted in the absence of any commercial or financial relationships that could be construed as a potential conflict of interest.

## Publisher’s Note

All claims expressed in this article are solely those of the authors and do not necessarily represent those of their affiliated organizations, or those of the publisher, the editors and the reviewers. Any product that may be evaluated in this article, or claim that may be made by its manufacturer, is not guaranteed or endorsed by the publisher.

## Footnotes

1
https://bitbucket.org/tasseladmin/tassel-5-source/wiki/Tassel5GBSv2Pipeline

2
https://www.rosaceae.org/Analysis/9450778

3
http://revigo.irb.hr
