# Supplementary material for: An Upgraded, Highly Saturated Linkage Map of Japanese Plum (Prunus salicina Lindl.), and Identification of a New Major Locus Controlling the Flavan-3-ol Composition in Fruits
Source: Front Plant Sci. 2022 Mar 4;13:805744. doi: 10.3389/fpls.2022.805744 (PMC8931734; doi:10.3389/fpls.2022.805744)
Supplement: Supplementary file 1 [file Table_1.DOCX]

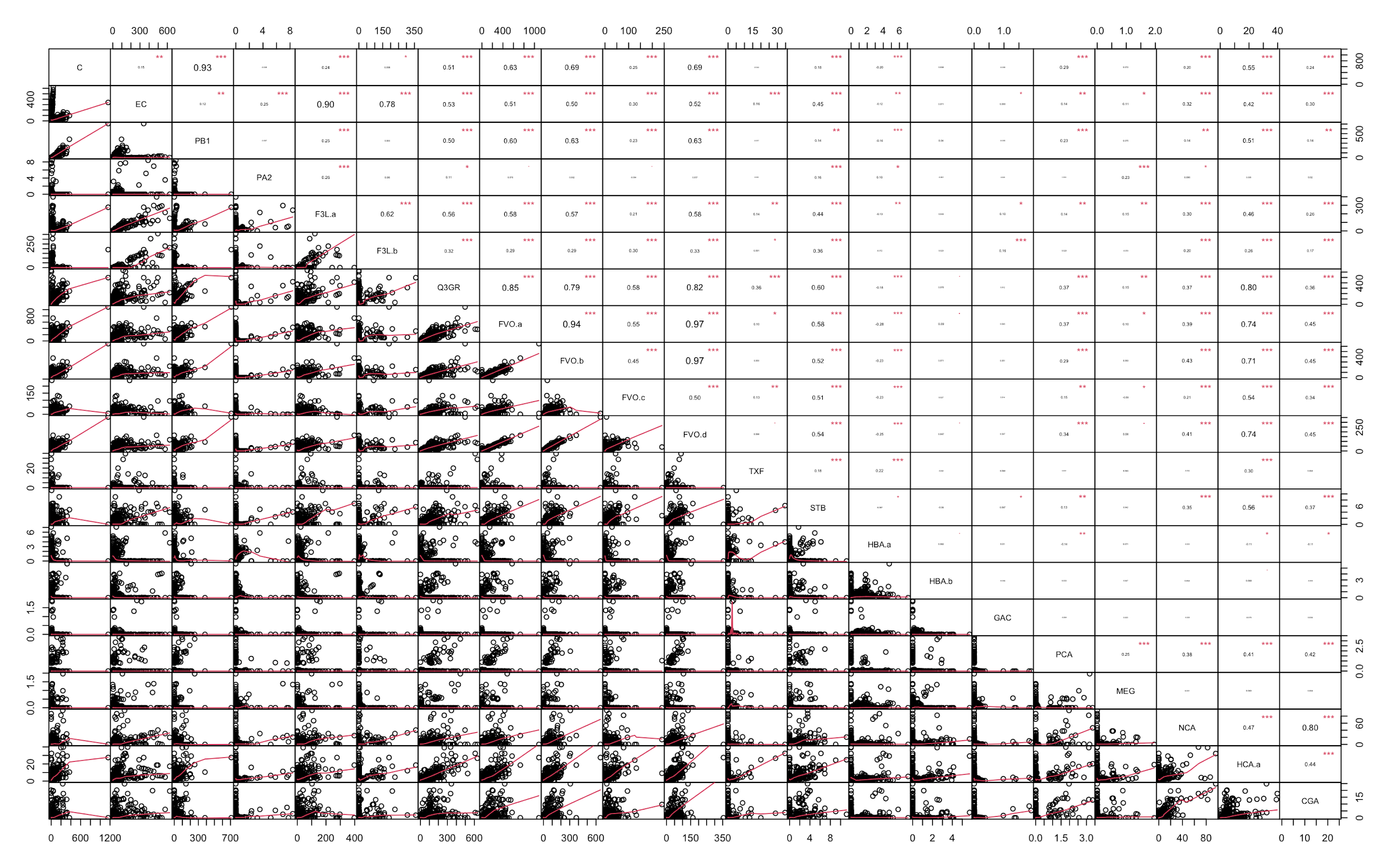


**Supplementary Figure 1.** Correlation matrix of phenolic compounds from an F1 population of Japanese plum fruits, identified through UHPLC DAD-Orbitrap-MS and quantified by HPLC-DAD. The size of the numbers in the top panel indicates a higher (bigger size) or lower (smaller size) correlation. C, catechin; CGA, chlorogenic acid; EC, epicatechin; F3L-a and F3L-b, unidentified flavan-3-ol dimers (a and b); FVO, unidentified flavonols (a – d); GAC, gallic acid; HBA, unidentified hydroxybenzoic acids (a and b); HCA, unidentified hydroxycinnamic acid (a); MEG, methyl gallate; NCA, neochlorogenic acid; PA2, proanthocyanidin A2; PB1, proanthocyanidin B1; PCA, protocatechuic acid; Q3GR, quercetin 3-glucoside + quercetin 3-rutinoside; STB, stilbene; TXF, taxifolin.


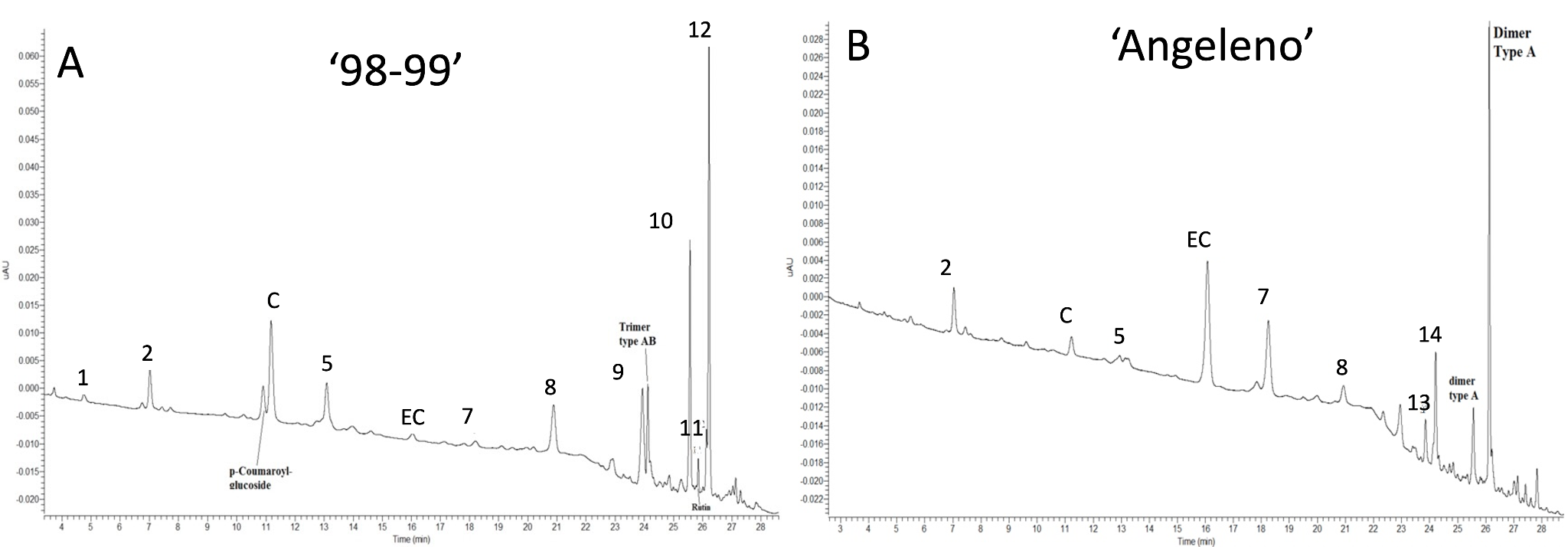


**Supplementary Figure 2.** HPLC-DAD profiles from the identified phenolic compounds in the cultivars ‘98-99’ (A) and ‘Angeleno’ (B). Axes represent the retention time (X-axis) and the absorbance at 275 nm (Y-axis). Numbers and acronyms correspond to: 1, protocatechuic acid 2, chlorogenic acid; 3, p-coumaric acid; C, catechin; 5, flavan-3-ol dimer type B (PB1); EC, epicatechin; 7, flavan-3-ol dimer type B; 8, dihydro caffeoylquinic acid; 9, taxifolin; 10, flavan-3-ol dimer type A (PA2); 11, quercetin-3-rutinoside; 12, quercetin-3-glucoside; 13, flavan-3-ol dimer type B; 14, flavan-3-ol trimer type AB.


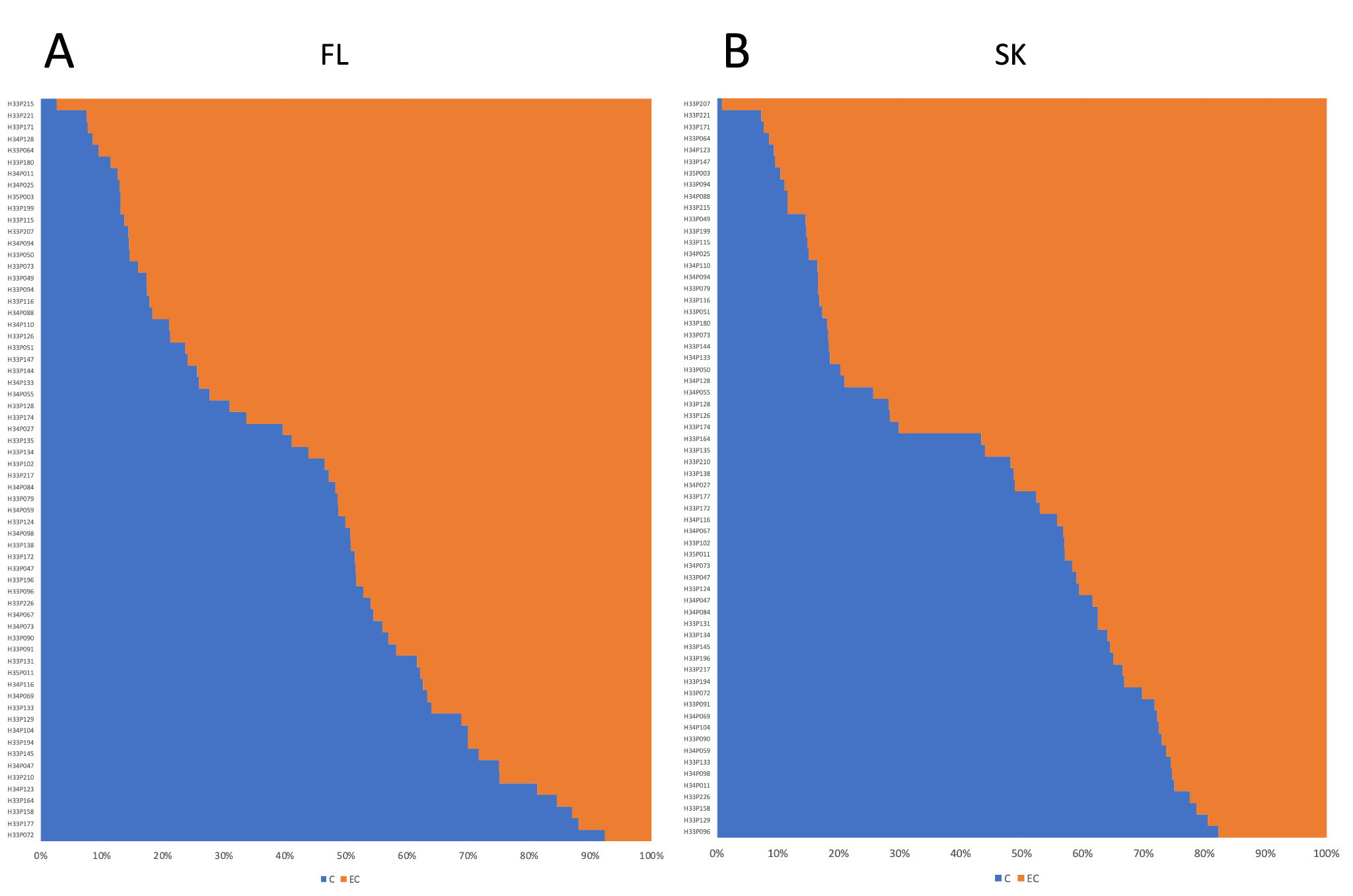


**Supplementary Figure 3.** Relative content of C and EC in flesh (A) and skin (B) of Japanese plum fruits from an F1 progeny (<‘98-99’ x ‘Angeleno’). Data correspond to the relative content of C and EC, measured by the HPLC-DAD technique. C, catechin; EC, epicatechin.
